# Molecular insights into an ancient form of Paget’s disease of bone

**DOI:** 10.1073/pnas.1820556116

**Published:** 2019-04-29

**Authors:** Barry Shaw, Carla L. Burrell, Darrell Green, Ana Navarro-Martinez, Daniel Scott, Anna Daroszewska, Rob van ’t Hof, Lynn Smith, Frank Hargrave, Sharad Mistry, Andrew Bottrill, Benedikt M. Kessler, Roman Fischer, Archana Singh, Tamas Dalmay, William D. Fraser, Kirstin Henneberger, Turi King, Silvia Gonzalez, Robert Layfield

**Affiliations:** ^a^School of Life Sciences, University of Nottingham, NG7 2UH Nottingham, United Kingdom;; ^b^Research Centre for Evolutionary Anthropology and Paleoecology, Liverpool John Moores University, L3 3AF Liverpool, United Kingdom;; ^c^Faculty of Archaeology, Leiden University, 2333 CC Leiden, The Netherlands;; ^d^Norwich Medical School, University of East Anglia, NR4 7TJ Norwich, United Kingdom;; ^e^Institute of Ageing and Chronic Disease, University of Liverpool, L7 8TX Liverpool, United Kingdom;; ^f^Department of Clinical Biochemistry and Metabolic Medicine, Royal Liverpool and Broadgreen University Hospitals National Health Service Trust, L7 8XP Liverpool, United Kingdom;; ^g^Department of Rheumatology, Royal Liverpool and Broadgreen University Hospitals National Health Service Trust, L7 8XP Liverpool, United Kingdom;; ^h^Norton Priory Museum and Gardens, WA7 1SX Runcorn, United Kingdom;; ^i^Protein & Nucleic Acid Chemistry Laboratory, University of Leicester, LE1 9HN Leicester, United Kingdom;; ^j^Target Discovery Institute, University of Oxford, OX3 7FZ Oxford, United Kingdom;; ^k^School of Biological Sciences, University of East Anglia, NR4 7TJ Norwich, United Kingdom;; ^l^Department of Clinical Biochemistry, Norfolk and Norwich University Hospital, NR4 7UY Norwich, United Kingdom;; ^m^Institute for Biochemistry and Biology, University of Potsdam, 14476 Potsdam, Germany;; ^n^Department of Genetics and Genome Biology, University of Leicester, LE1 7RH Leicester, United Kingdom

**Keywords:** Paget’s disease, osteosarcoma, p62, SQSTM1, paleoproteomic

## Abstract

Paget’s disease of bone (PDB) is a chronic skeletal disorder that can affect one or several bones in individuals older than 55 y of age. PDB-like changes have been reported in archaeological remains as old as Roman, although accurate diagnosis and natural history of the disease is lacking. Six skeletons from a collection of 130 excavated at Norton Priory in the North West of England, which dates to medieval times, show atypical and extensive pathological changes resembling contemporary PDB affecting as many as 75% of individual skeletons. Disease prevalence in the remaining collection is high, at least 16% of adults, with age at death estimations as low as 35 y. Despite these atypical features, paleoproteomic analysis identified sequestosome 1 (SQSTM1) or p62, a protein central to the pathological milieu of PDB, as one of the few noncollagenous human sequences preserved in skeletal samples. Targeted proteomic analysis detected >60% of the ancient p62 primary sequence, with Western blotting indicating p62 abnormalities, including in dentition. Direct sequencing of ancient DNA excluded contemporary PDB-associated *SQSTM1* mutations. Our observations indicate that the ancient p62 protein is likely modified within its C-terminal ubiquitin-associated domain. Ancient miRNAs were remarkably preserved in an osteosarcoma from a skeleton with extensive disease, with miR-16 expression consistent with that reported in contemporary PDB-associated bone tumors. Our work displays the use of proteomics to inform diagnosis of ancient diseases such as atypical PDB, which has unusual features presumably potentiated by yet-unidentified environmental or genetic factors.

## Significance

We identify an ancient and atypical form of Paget’s disease of bone (PDB) in a collection of medieval skeletons exhibiting unusually extensive pathological changes, high disease prevalence, and low age-at-death estimations. Proteomic analysis of ancient bone-preserved proteins combined with analysis of small RNAs supports a retrospective diagnosis of PDB. Remains affected by other skeletal disorders may therefore hold a chemical memory amenable to similar molecular interrogation. Abnormalities in a contemporary PDB-linked protein detected in ancient tooth samples indicate that dentition may represent an unexplored storehouse for the study of skeletal disorders. Our work provides insights into the natural history of PDB and prompts a similar revaluation of other archaeological collections.

Paget’s disease of bone (PDB) is the second most common metabolic bone disorder (1). The condition is characterized by focal abnormalities in bone remodeling. Contemporary PDB typically affects one or several bones in individuals older than 55 y of age (2). Pathogenesis involves three phases, starting with a lytic phase in which bone turnover is markedly increased and increased resorption occurs through the action of abnormal hypernucleated osteoclasts (3). A second mixed phase of lytic and osteoblastic activity is dominated by rapid increases in osteoblast activity giving rise to abnormal bone with irregular deposition of collagen fibers. Finally, in a sclerotic phase, bone formation predominates, with newly formed bone being disorganized (woven) and mechanically weaker, overall resulting in skeletal lesions with abnormal architecture and predisposition to fracture. PDB commonly affects the femur, tibia, pelvis, skull, and spine but is rarely seen in non–weight-bearing bones such as the fibula (4). Affected individuals can experience complications such as bone pain, bone deformity, nerve compression syndromes, and susceptibility to fractures. Osteosarcoma is a rare but severe complication (1).

Western Europe is thought to be the origin of PDB, specifically the United Kingdom (5). Populations of British descent report the highest prevalence, currently 1–2% in individuals older than 55 y (6). The North West of England represents a particular hotspot, whereas PDB is rare in Scandinavia, Asia, and Africa (1). Incidence and severity of newly diagnosed cases has decreased; for example, the incidence of PDB in the city of Lancaster has decreased from 8.3% in the 1970s to 0.8% at present (7, 8). Such secular changes suggest a role for currently undefined but important changes in environmental factors that affect genetically predisposed individuals (1, 8). Infection with paramyxoviruses such as measles, supraphysiological biomechanical loading, and other environmental triggers have been considered, but evidence is inconclusive (1).

Genetic factors in the etiology of PDB are well defined. As many as 40% of affected individuals have relatives with PDB. Susceptibility is determined by driver mutations in genes relevant to osteoclast function, which include colony-stimulating factor 1 (*CSF1*), dendrocyte-expressed seven transmembrane protein (*DCSTAMP*), TNF receptor superfamily member 11a (*TNFRSF11A*), TNF receptor superfamily member 11b (*TNFRSF11B*), optineurin (*OPTN*), nucleoporin 205 (*NUP205*), Ras and Rab interactor 3 (*RIN3*), and promyelocytic leukemia (*PML*) (1, 6). As many as 50% of familial cases, as well as a smaller number of sporadic cases, carry heterozygous mutations affecting the sequestosome 1 (*SQSTM1*) gene that encodes the SQSTM1 or p62 protein. Such variants impact p62 in a domain-specific manner, generally being associated with missense and truncating mutations within the C-terminal ubiquitin-associated (UBA) domain (residues 387–436) (9, 10). Patients with *SQSTM1* mutations are typically diagnosed earlier than those without, with mutation status alone playing a major role in determining the disease phenotype in patients (11, 12). Mice with a proline-to-leucine mutation at codon 394 of *sqstm1*, equivalent to the most common P392L mutation in humans, develop an age-associated bone disorder with similarity to PDB (13). This bone disorder can be prevented by infusion with zoledronic acid, which is the first-line treatment for PDB in humans (14). *SQSTM1* is also relevant to disease etiology in PDB patients without mutations. For example, p62 is overexpressed in patient-derived cells regardless of mutation status, and p62 immunoreactivity is a feature of the nuclear inclusion bodies that characterize pagetic osteoclasts (15, 16). miRNA dysregulation has also been shown to play a role, with down-regulation of miR-16 observed in PDB (17). Increased miR-16 expression was also observed in PDB-associated osteosarcoma (PDB-OS) compared with nontransformed pagetic bone lesions (17). MiR-16 is a negative regulator of the *SQSTM1* transcript (18).

PDB-like features based on macroscopic and radiographic changes resembling those in the contemporary disorder have been reported in archaeological remains dating as far back as Roman, with the highest number of cases identified as medieval (1066–1538 AD) (19). Norton Priory in the North West of England is one of the most excavated monastic sites in Europe, with a collection of 130 skeletons dating to the medieval period. A previous review of the collection identified pathological changes resembling PDB in six individuals, ∼5% of the adult sample, which is more than double than in comparable excavations in the North East of England (20). Our reanalysis indicated that skeletal involvement in the six cases at Norton Priory is more extensive than previously reported, raising questions about whether the disorder was a form of ancient PDB or another bone disease.

Paleoproteomics is a relatively new field that has demonstrated that proteins, typically abundant structural proteins such as collagens, resist taphonomy and persist in mineralized tissues over many years (21). MS-based protein sequencing provides insights into species identification and evolutionary relationships and potentially helps to identify human disease, especially in conditions in which ancient DNA may be degraded. Paleoproteomic studies focus on the most abundant bone proteins such as collagen; however, noncollagen protein sequences can also be recovered from archaeological samples. For example, peptide sequences from more than 100 different proteins were recovered from a 43,000-y-old mammoth femur (22). Western blotting detected prostate-specific antigen in bone from an ancient specimen with metastatic prostate cancer (23). Proteomic analysis of a 2,000-y-old bone tumor identified several contemporary biomarkers (24). These proteins contain an additional level of information that offers opportunities for exploring the history and evolution of disease, potentially even through the detection of mutations or modifications within protein sequences (25).

We reasoned that bone-specific proteomes might provide insights into the skeletal disorder at Norton Priory. We catalog the highly unusual phenotypic features of the bone disorder within the Norton Priory collection, highlighting similarities and important differences vs. contemporary forms of PDB. We show that paleoproteomic analysis (sequencing of ancient p62 protein) and analysis of ancient small RNAs can be used to support the diagnosis of a PDB-like disorder several hundred years after death.

## Results

### Atypical Features of a PDB-Like Disorder at Norton Priory.

**Fig. 1.**
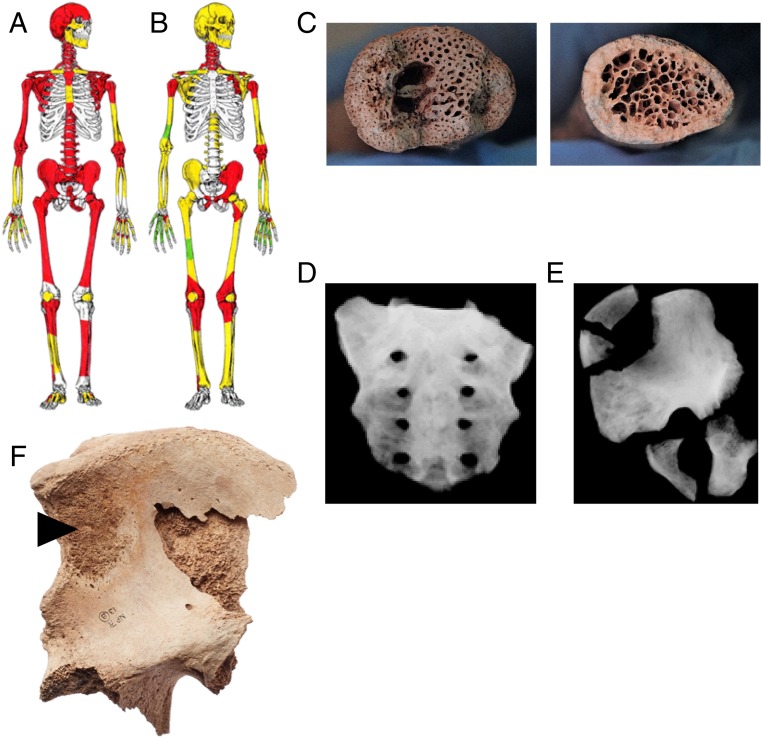
Skeletal distribution of macroscopic changes (red), internal lytic changes identified by radiographic analysis (yellow), and unaffected bones (green) of a PDB-like disorder in SK101 (*A*) and SK29 (*B*). (*C*) Macroscopic observation of internal structural changes in the right clavicle of SK37 (*Left*) compared with the normal cortical and trabecular structure of an unaffected right clavicle (*Right*; SK50). Radiographic imaging of SK37 sacrum (*D*) and hip (*E*). (*F*) Macroscopic observation of osteosarcoma (arrowhead) in the pelvis of SK29. The extracortical portion of the tumor exhibits a slight radiant alignment of bone that is often referred to as a “sunburst” appearance. Reprinted with permission of the Norton Priory Museum Trust.

A review of six adult skeletons from the Norton Priory collection (130 articulated skeletons, 114 of which are adult) identified macroscopic changes resembling contemporary PDB (20). Reanalysis using radiography indicates that the disease in the six cases is unusually extensive (*SI Appendix*, Table S1). For example, SK101 is an adult male assessed at 45–49 y of age dated to the 15th century and is one of the most extensively affected individuals, with PDB-like changes involving 75% of his skeleton (Fig. 1*A*). SK29 is also markedly affected with PDB-like pathology (75% of the skeleton), although, in this case, most changes were detected only by radiography (Fig. 1*B*). SK29 exhibits an osteosarcoma to both os coxae (Fig. 1*F*) (20). Accelerator MS (AMS) radiocarbon dating and stable isotopic analysis (C, N, Sr, and O) indicated that all six skeletons are medieval in age (1050–1390 AD), had a mainly marine-based diet, and identified as local to the North West of England (*SI Appendix*, Tables S2 and S3).

We extended the study of six skeletons to include additional remains in the collection. We analyzed in detail a further 12 skeletons that displayed visible lesions similar to contemporary PDB. Each of the 12 skeletons were subjected to complete macroscopic and radiographic review with additional AMS radiocarbon dating and isotope analysis. SK37, an adult male assessed at 30–35 y of age, shows distinct porous macroscopic changes involving 40% of his skeleton (Fig. 1*C*). Internal structural changes of the bone disease can be clearly observed in his right clavicle that were not evident in a comparable sample from an unaffected individual (Fig. 1*C*). Radiographic imaging identified PDB-like lesions affecting 75% of the affected skeleton, including thickened and enlarged bone in the sacrum (Fig. 1*D*) and os coxae (Fig. 1*E*).

Radiocarbon analysis confirmed that the additional 12 skeletons are also medieval in age, with the combined 18 skeletons covering 450 y of Norton Priory’s history (1020–1479 AD). Some individuals appear to have lived during the same period of time (*SI Appendix*, Table S2). Carbon (^13^C) and nitrogen (^15^N) stable isotope analysis for the additional 12 skeletons identified high levels of nitrogen, again revealing a marine-based diet, which is typical of a monastic lifestyle (*SI Appendix*, Table S3). Consistent with the slight male predilection seen in contemporary PDB, there was higher prevalence of affected males (*n* = 15) than females (*n* = 3) in the 18 skeletons. Norton Priory is a monastic site that presents a bias toward adult males overall (male, *n* = 85; female, *n* = 29). Pathological changes were extensive in all of the additional 12 skeletons, with 40% of each skeleton displaying macroscopic PDB-like lesions. Radiographic analysis highlighted that 75% of the skeleton was affected in some cases. Identification of 18 medieval skeletons with PDB-like lesions creates a new population prevalence of 14% (18 of 130), or 16% of the adult sample (18 of 114) at Norton Priory. Overall age at death assessment was 35–59 y of age, including 13 of 18 individuals under the age of 50 y (*SI Appendix*, Table S2). Compared with contemporary PDB, the skeletal disorder at Norton Priory is therefore characterized by unusually extensive pathology and high prevalence with relatively low estimated age at death.

Skeletal distribution of disease in the 18 skeletons generally follows contemporary distribution, i.e., femur, pelvis, skull, and spine (*SI Appendix*, Table S4). Involvement of the fibulae is rarely reported in contemporary PDB, but here it is one of the most commonly affected bones. Complications reported in patients with contemporary PDB include osteoarthritis, deafness, fractures, deformity, and malignancy. In the Norton Priory skeletons, very few complications are observed, with a lack of evidence of deformity in weight-bearing bones (e.g., tibia and/or femur) or pseudofractures. Malignant transformation occurs in 1:1,000 of contemporary PDB patients. SK29 harbors an osteosarcoma in the pelvis, which is particularly rare for the archaeological record (Fig. 1*F*). Although skeletal changes in the Norton Priory remains broadly resemble those seen in contemporary PDB, there are many atypical features within the Norton Priory remains (*SI Appendix*, Table S4).

### Abnormal SQSTM1 (p62) Is Preserved in Skeletal Samples from the PBD-Like Disorder.

**Fig. 2.**
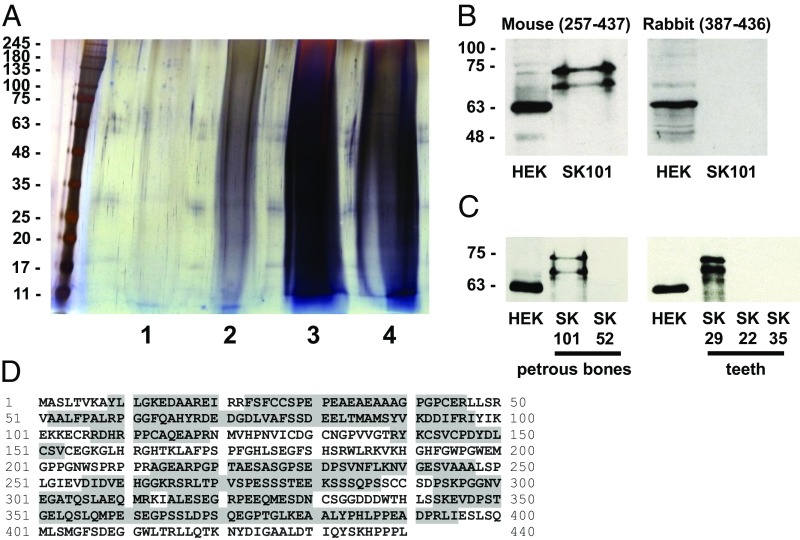
Proteomic analysis of ancient skeletal samples. (*A*) SDS/PAGE and silver stain analysis of sequential fractions of proteins extracted from SK101 petrous bone, with molecular weight markers (in kilodaltons) shown to the left. Lane 1 is guanidine-HCl extract. Lane 2 is guanidine-HCl/EDTA extract. Lane 3 is phosphate buffer/EDTA extract. Lane 4 is insoluble pellet. In each lane, 10% of total protein extracted from 50 mg bone was loaded. (*B*) Western blot of insoluble pellet fraction (from lane 4) using mouse and rabbit anti-p62 antibodies, with HEK293T cell extract as positive control. (*C*) Western blot of SK101 and SK52 petrous bone using mouse anti-p62 antibodies, plus Western blot of SK29, SK22, and SK35 teeth extract (insoluble pellet) using mouse anti-p62 antibodies, with HEK293T cell extract as positive control. (*D*) Combined MS data indicating WT ancient human p62 protein sequence detected (shaded) from SK101 petrous (insoluble pellet) fraction.

We performed molecular diagnosis of the reported bone disorder. We reasoned that bone-specific proteomes might provide insights into disease etiology. For our paleoproteomic analysis, we accessed a sample of petrous bone (part of the temporal bone) from one of the originally described six skeletons. SK101 shows extensive macroscopic and radiographic evidence of disease, with most bones affected (Fig. 1*A* and *SI Appendix*, Table S1). The cranium was among the most highly affected areas. Extracts from the petrous sample revealed complex protein patterns on SDS/PAGE with a high degree of smearing (Fig. 2*A*). Complete protein extracts from different fractions were subject to LC-MS/MS to catalog constituent proteins, including the insoluble pellet fraction resulting after buffer extractions of the bone matrix (Fig. 2*A*). A total of 19 human proteins were detected with a minimum of two peptide sequences within this pellet fraction. The majority of the identified proteins were collagens and cytoskeletal components (*SI Appendix*, Table S5). Peptide sequences matching to human p62 (8% sequence coverage) were detected in the pellet fraction from three exclusive unique peptides. This preferential preservation of p62—a protein not previously reported in paleoproteomic analysis to our knowledge—in SK101 petrous suggests that the associated disorder was likely an ancient form of PDB in this individual.

We also performed Western blots using the pellet fraction from SK101 petrous with mouse anti-p62 antibodies. Two strongly immunoreactive bands were detected, migrating with higher molecular weight than a ∼62-kDa control band from a human cell line (Fig. 2*B*). Weaker immunoreactivity corresponding to these higher molecular weight forms of ancient p62 was also detected in the TCA-precipitated phosphate extract from SK101 petrous (Fig. 2*A*). Ancient p62 was not detected by a rabbit polyclonal antibody raised against the C-terminal UBA domain of the human protein (residues 387–436), although this antibody also readily detected a control ∼62-kDa band from human cells (Fig. 2*B*). To determine if abnormally migrating p62 protein was a common feature of other skeletons in the collection, we expanded our Western blotting with the mouse antibody to include four additional samples from the original six skeletons. For one of these skeletons, SK52, a sample of petrous bone was available. For the other three skeletons (SK22, SK29, SK35), we sampled teeth. Although petrous from SK52 was p62-negative, a tooth sample from SK29 produced an equivalent immunoreactive profile to SK101, with the two abnormally migrating higher molecular weight forms of ancient p62 readily detected (Fig. 2*C* and Table 1).

**Table 1.** Paget’s-like changes, p62 reactivity and endogenous DNA in bone and tooth samples

Skeleton	Macroscopic/radiographic Paget’s-like changes	Samples (p62 reactivity*)	Samples (endogenous DNA^†^)
SK101	Yes^‡^	Petrous (+), Femur (−)	Petrous (32.1%)
SK52	Yes	Petrous (−), Femur (−)	Petrous (47.2%)
SK29	Yes	Tooth (+)	Tooth (4.1%)
SK22	Yes	Tooth (−)	Tooth (26.8%)
SK35	Yes	Tooth (−), Femur (+)	Tooth (1.3%)
SK55	Yes	—	Rib (0.0004%)
SK32	Yes	Petrous (−)	—
SK37	Yes	Petrous (+)	—
SK27	No	Petrous (−)	—
SK28	No	Petrous (−)	—

(+), positive; (−), negative; —, not tested.

* p62 immunoreactivity in the indicated samples was assessed by Western blotting with mouse-anti-p62 antibodies.

^†^ Endogenous DNA content for different skeletal samples as indicated.

^‡^ Indicates clear Paget’s like macroscopic and/or radiographic changes in bone.

The rabbit antibody also failed to detect ancient p62 from the tooth of SK29. Positive detection of p62 with the mouse antibody in teeth is unexpected, as pagetic changes, although reported to affect the jaw, have rarely been reported in dental pulp. This finding presumably represents involvement of odontoclasts. Previous dental pulp paleoproteomic analysis did not detect p62 protein sequences (26, 27). Blotting was expanded to petrous samples from an additional two cases within the collection that showed evidence of disease (SK32, SK37) plus two cases with no obvious pathology (SK27, SK28). We detected weak p62 reactivity using the mouse antibody in the insoluble pellet fraction of SK37 (Table 1). We were also able to analyze insoluble pellet fractions from femur samples of three of the original six skeletons (SK101, SK52, SK35). Although SK101 petrous bone was previously found to be p62-positive (Fig. 2*C*), the corresponding femur sample was negative (Table 1). In contrast, SK35 femur was found to be p62-positive (Table 1) despite the corresponding tooth sample being negative (Fig. 2*C*). A total of four of seven skeletons with evidence of PDB-like changes showed p62 immunoreactivity by Western blot with mouse anti-p62 in at least one sample tested. Both skeletons without evidence of pathology showed no immunoreactivity (Table 1). We speculate that the variability of ancient p62 detection in the affected skeletons likely reflects differences in protein preservation resulting from varied burial conditions. Inadvertent sampling of skeletal areas with different degrees of disease activity may also be a factor, as it was not feasible to radiographically assess all bone samples before drilling.

### Primary Sequence of the Ancient p62 Protein.

LC-MS/MS shotgun analysis using the linear trap quadrupole (LTQ) Orbitrap Velos mass spectrometer with tryptic digest detected three unique peptide sequences corresponding to human p62 (*SI Appendix*, Table S5). To provide additional protein sequence data, we extended this analysis to SDS/PAGE gel slices limited to an area that included both of the p62 immunoreactive bands from SK101. Separation and individual identification of the bands was not achieved as a result of masking by the protein smear (Fig. 2*A*). Peptide sequencing was performed by using the LTQ Orbitrap Velos with tryptic digestion (*SI Appendix*, Fig. S1*A*) and an Orbitrap Fusion Lumos mass spectrometer with elastase digestion (*SI Appendix*, Fig. S1*B*). Many more peptides from the 440-residue human p62 sequence were identified, which yielded a total combined sequence coverage of 60% (266 of 440 residues; Fig. 2*D*). Comparable analysis of SK29 tooth samples also detected 10% of the human p62 sequence with no additional coverage compared with SK101. Error-tolerant searches provided no evidence of any p62 sequence variants. The combined MS analyses confirmed that the ancient p62 sequence is WT and intact between residues 342 and 395 but did not detect any protein sequence beyond I395, the region corresponding to the majority of the C-terminal UBA domain (*SI Appendix*, Fig. S2). This is the region of p62 removed by a PDB-associated E396X truncating mutation known to be associated with an early age at onset (∼47 y) and high number of bones affected (∼6) in contemporary PDB patients (10).

### Sequencing of Ancient DNA.

Targeted proteomics directed cDNA sequencing to investigate the possible presence of the E396X mutation (T insertion at +1225). For ancient DNA analysis, petrous bone or tooth samples were sourced from the original six skeletons. For SK55 (not tested in the blotting study), an additional sample of rib bone was available. Samples were cleaned and UV-treated before sequencing yielded estimates of endogenous DNA (Table 1). These values ranged from 47% (SK52 petrous) to 0.0004% (SK55 rib) with average read lengths of 50 bp and damage patterns consistent with DNA extracted from ancient samples. There was no obvious correlation between quality of ancient DNA and detection of p62 by Western blot in corresponding samples.

**Fig. 3.**
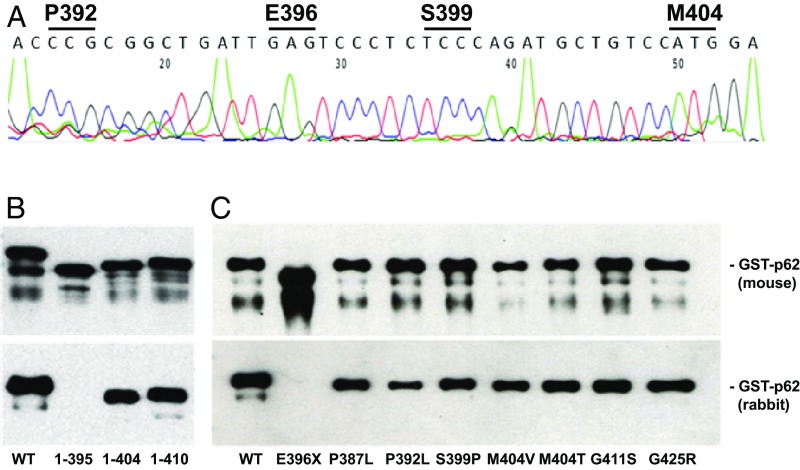
Apparent absence of mutations in ancient p62. (*A*) Representative targeted DNA sequencing of ancient *SQSTM1*, in this case corresponding to residues 392–404 of human p62. Presence of WT codons at known PDB mutation sites are indicated. (*B*) Western blotting using recombinant human GST-p62 protein sequences as indicated. WT represents full-length WT 440-residue sequence. Mouse and rabbit anti-p62 antibodies located an epitope for the latter between residues 396 and 404. (*C*) Western blot detection of human GST-p62 protein sequences with PDB mutations as indicated, using mouse and rabbit anti-p62 antibodies.

Given the degraded nature of ancient DNA and varying degrees of endogenous DNA preservation, primers were designed to create an amplicon with a maximum size of 70–80 bp covering the genomic region that includes residues 392–404 of p62. An additional set of pigtail primers were designed to allow for clearer direct sequencing. Sequencing was successful for five of six samples (Fig. 3*A*). In all cases, only WT *SQSTM1* sequence was identified, including nucleotides corresponding to P392–E396, which is consistent with protein sequencing (Fig. 2*D*). There was no evidence of insertion or deletion. The contemporary PDB-associated E396X truncating mutation, as well as P392L, S399P, and M404V/T missense mutations that map to this region, were excluded (*SI Appendix*, Fig. S2).

### Western Blot Insights into Ancient p62 Sequences.

The failure of the rabbit antibody raised against residues 387–436, i.e., the UBA domain, to detect ancient p62 supported the possible mutation/deletion of *SQSTM1* or modification of a corresponding rabbit epitope (Fig. 2*B*). To understand the primary structure of the ancient p62 protein, we fine-mapped the rabbit antibody epitope. C-terminal deletion analysis showed that the rabbit antibody was able to detect a recombinant p62 (1–404) sequence but not p62 (1–395), which is equivalent to the E396X mutant (Fig. 3*B*). We pinpointed an epitope between residues 396 and 404 (*SI Appendix*, Fig. S2). PDB-associated variants located within this epitope (S399P, M404V, M404T) that are generated as GST-fusion proteins in *Escherichia coli* were detected by the rabbit antibody, further excluding mutation of this region as the reason for failed detection of ancient p62 by the antibody. Other common missense mutations including P387L, P392L, G411S, and G425R were included in the panel (*SI Appendix*, Fig. S2). The mouse and rabbit antibodies successfully recognized all missense mutants tested (Fig. 3*C*). The PDB-associated E396X truncating mutant equivalent to the p62 (1–395) protein sequence used in the epitope mapping that removes the majority of the UBA domain was not recognized by the rabbit antibody (Fig. 3*C*). This mutation had already been excluded by DNA sequencing. We conclude that the failure to detect ancient p62 with the rabbit antibody where the mouse antibody gave reactivity is unlikely to be caused by a mutation affecting the rabbit epitope (residues 396–404) with corresponding DNA being intact. Rather, failure to detect is likely a result of epitope masking by taphonomic changes that are diagenetic in origin, or there is an undefined disease-associated posttranslational modification(s) of the ancient p62 protein that is a feature of the disorder.

### Analysis of Ancient Small RNAs in Osteosarcoma.

**Fig. 4.**
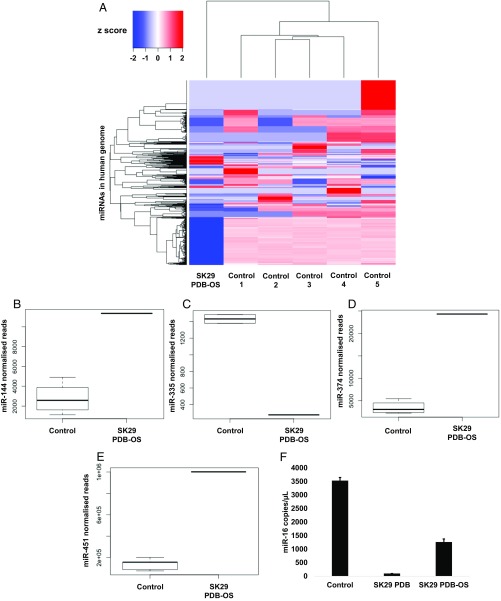
Ancient miRNA analysis in SK29, which harbors an osteosarcoma in the pelvis. (*A*) Hierarchical cluster analysis of differentially expressed miRNAs (*y* axis) between contemporary controls and SK29 PDB-OS (*x*-axis). *Z*-score refers to high (red) and low (blue) miRNA expression using normalized values compared with the mean of total sequencing reads. (*B*) Box plot of controls vs. SK29 PDB-OS shows that miR-144 is highly expressed in SK29 PDB-OS. (*C*) Box plot of controls vs. SK29 PDB-OS shows that miR-335 is down-regulated in SK29 PDB-OS. (*D*) Box plot of controls vs. SK29 PDB-OS shows that miR-374 is highly expressed in SK29 PDB-OS. (*E*) Box plot of controls vs. SK29 PDB-OS shows that miR-451 is highly expressed in SK29 PDB-OS. (*F*) Digital PCR for miR-16 expression performed with pooled cDNA obtained from contemporary controls, cDNA obtained from a PDB lesion in SK29, and cDNA obtained from PDB-OS in SK29. Data are reported as copies per microliter as calculated by Poisson distribution. Error bars represent SD. Consistent with previous data, miR-16 is highly expressed in PDB-OS compared with nontransformed PDB.

A rare complication of PDB is malignant transformation. We recently reported changes in miRNAs in PDB-OS. We detected a high expression of miR-16 in PDB-OS compared with samples from nontransformed pagetic bone lesions, indicating that miR-16 may serve as a biomarker of malignant transformation (17). One of the p62-positive skeletons, SK29, shows osteosarcoma on both sides of the pelvis (Fig. 1*F*). This finding provided an opportunity to explore ancient miRNAs. Next-generation sequencing obtained 6.6 million reads in the SK29 osteosarcoma. Mapping against the human genome and corresponding annotations showed clustering of miRNA expression in noncancerous controls compared with SK29 osteosarcoma (Fig. 4*A*). This quality-control step is consistent with previous small RNA sequencing studies reporting controls and disease. Of the 3.7% genome matching reads in the SK29 osteosarcoma, 71% mapped to miRNA loci and 0.5% mapped to tRNA loci. Differential expression analysis showed a high expression of miR-144 (Fig. 4*B*), a low expression of miR-335 (Fig. 4*C*), a high expression of miR-374 (Fig. 4*D*), and a high expression of miR-451 in SK29 osteosarcoma. Quantitative differential expression analysis showed that miR-16 was highly expressed in the ancient osteosarcoma compared with a nontransformed femoral PDB lesion from the same individual (Fig. 4*F*). To the best of our knowledge, this is the first demonstration of successful extraction of ancient miRNAs and tRNAs from archaeological bone samples.

## Discussion

Several of our observations support the notion that Norton Priory in the North West of England was a medieval hotspot for an atypical form of PDB. This bone disorder presumably represents a variant of contemporary PDB. There are considerable phenotypic differences associated with the Norton Priory remains compared with modern-day PDB. These differences include extensive disease (75% of bones in some affected skeletons), low age-at-death estimations (13 of 18 individuals younger than 50 y), and high disease prevalence in the adult sample (16%), but little evidence of bone deformities. Macroscopic and radiographic analysis combined with characteristic pathological changes and molecular signatures support a retrospective diagnosis of an atypical form of PDB. Internal structural changes of bones from the collection are consistent with those expected for PDB, with macroscopic thickening and enlargement of bone, as is the presence of an osteosarcoma in one of the affected skeletons. Within this osteosarcoma, increased miR-16 expression is consistent with that reported in contemporary PDB (17).

Detection of ancient p62 as one of the few noncollagenous proteins in skeletal samples (bones and teeth) based on a combination of peptide sequencing and Western blotting is strongly indicative of a diagnosis of PDB, not least because, to the best of our knowledge, no other paleoproteomic analysis has detected this PDB-associated protein. Although protein retrieval is buffer- and protocol-dependent, we extensively reviewed published studies describing proteomics of ancient bone and teeth, including those that used comparable methods to ours, and found no previous reports of detection of ancient p62 protein. In our study, p62 protein sequences were detected by using two different mass spectrometers in unconnected laboratories from more than one tissue sample, in extracts generated by several investigators on different occasions. Unlike ancient DNA analysis, protein detection does not rely on amplification, countering the possibility of contamination of samples. Our findings are highly significant, as they represent one of the first molecular diagnoses of an ancient disorder based largely on protein sequence-based methods. Those methods indicate that ancient remains affected by other skeletal disorders may also hold a “chemical memory” that is amenable to proteomic interrogation.

Several speculations remain regarding the detection and observed properties of the ancient p62 protein. p62 was variably detected, having been observed in only 4 of 10 samples tested from 4 of 7 affected individuals. We consider that a combination of differential preservation of the skeletons throughout burial with the fact that, although disease was extensive, not all samples tested may have been affected by pagetic changes may be an explanation. PDB is principally an osteoclast disorder, with p62 pathology being a feature of this cell type (16). Osteoclasts are rare in human bones that are unaffected by PDB.

p62 was detected in tooth samples even though PDB is not classically associated with involvement of dentition. Pagetic changes have been reported in teeth in rare cases of the disorder (28, 29). Roles for p62 in the regulation of autophagy in human dental pulp cells have also been described (30). It is possible that the atypical and extensive nature of this ancient form of PDB accounts for pathological accumulation of p62 protein in dentition. It is tempting to speculate that ancient and even contemporary teeth may more broadly act as a “storehouse” for the study of human skeletal disorders.

Ancient p62 survived burial and was well preserved for hundreds of years despite the fact that it is a simple intracellular scaffold protein, rather than an abundant and intractable structural protein such as collagen. We found enrichment of ancient p62 in the insoluble pellet fraction remaining after the standard buffer extractions. The characteristic p62-positive inclusions noted in PDB osteoclasts have previously been described based on EM to have organized viral-like paracrystalline morphology, which may underlie enhanced stability of the protein in the pagetic state (31). At the molecular level, p62 can form oligomeric filamentous structures and can undergo phase separation to form droplets with liquid-like properties (32, 33). These properties may again relate to p62 stability and be enhanced in the pagetic state, in which the protein is overexpressed (15). Accumulation of protein aggregates within inclusion bodies is a common feature of a range of neurodegenerative disorders with disease-associated misfolded proteins deposited in insoluble forms in the nucleus and cytoplasm (34). Ancient PDB may similarly represent a p62 “proteinopathy.”

Ancient p62 aberrantly migrates on SDS/PAGE–Western blot, with two prominent bands differentially detected by antibodies directed against different epitopes and with higher than expected denatured molecular weight. Sequencing of ancient DNA and blotting studies of recombinant mutant proteins with the different antibodies excludes common disease-associated missense mutations, although these would not be expected to account for the observed aberrant migration of the protein. Targeted DNA sequencing did not provide any evidence for gross genomic alterations within the p62 UBA domain, at least in the region encoding residues 392–404. In our proteomic analysis, we found no evidence of peptide sequences supportive of this alteration or of aberrant splicing. Although PDB-associated splice site mutations have been identified that give rise to shorter p62 sequences, the genomic region corresponding to and around residue E396 does not appear to harbor cryptic splice sites. Cryptic splicing events of *SQSTM1* cannot be completely discounted, such as splicing imparted by other genetic variants (10, 35, 36). We believe altered properties of the ancient p62 are most likely a result of taphonomic changes in the protein or some form of (possibly disease-related) posttranslational modification(s). Regarding taphonomic changes, from scrutiny of proteomic data, we found no evidence that the ancient p62 protein was heavily oxidized and/or deamidated. There are various reports in the literature of SDS-stable higher molecular weight forms of p62 with little comment on its nature or biological significance, which variously conclude that exposure to cigarette smoke, UV light, or cellular aging can induce covalent p62 oligomers (37). Significant discrete mass shift (as much as ∼10 kDa per modification) can occur as a result of covalent linkage with ubiquitin-like modifiers that could also mask antibody epitopes. Our proteomic data provided no evidence of ubiquitin, SUMO, or NEDD8 sequences in p62-containing fractions, and Western blots failed to detect ubiquitin immunoreactivity. Other covalent modifications such as cross-links via transglutamination cannot be discounted, and other contemporary genetic abnormalities could be at play.

Aside from involvement of and alterations in the ancient p62 protein, we also detected ancient miRNAs and tRNAs from skeletal samples. In line with our recent analysis of contemporary PDB-OS, miR-16 was highly expressed in samples extracted from an ancient osteosarcoma compared with extracts from a nontransformed PDB lesion (17). p62 serves as a signaling hub that can regulate various pathways relevant to cancer biology. Previous studies have demonstrated that the *SQSTM1* transcript that encodes p62 is negatively regulated by miR-16 (18). miR-16 expression changes in PDB-OS may not only represent a biomarker but may be directly relevant to tumorigenesis. It is salient to note that miRNAs are 10 times more stable than mRNA transcripts, and, unlike DNA, RNA is less prone to depurination, suggesting that dry environments may reduce the rate of degradation (38–40). Where ancient DNA and protein sequencing produces inconclusive data in archaeological studies, we suggest investigation of miRNAs.

Ancient PDB at Norton Priory is phenotypically unusual and apparently unique in the North West of England. Ancient PDB required radiographic analysis to confirm its presence/extent in many cases. It is possible that cases of PDB in other medieval skeletal collections have been overlooked, and a closer reevaluation of these is now merited. Although, as in many populations, the incidence of PDB has significantly decreased in the North West of England in the past few decades, this region has traditionally represented a hotspot for the disorder, suggesting that changing local environmental factors may represent a major but unexplained trigger. Strontium and oxygen isotope analysis has identified that at least five of the skeletons examined in our study are local to this area, supporting this modern etiology. It is also possible that the high prevalence of PDB in the Norton Priory collection was the result of accumulation of cases from a wider distribution as a result of the priory being seen as a “referral center” for the management of painful bones. Additional Sr and O isotope analysis is required to test this hypothesis.

Some studies have suggested a role for paramyxoviruses in PDB etiology, supported by the presence of virus-like nuclear inclusions in pagetic osteoclasts (41). Other studies have failed to confirm these findings and have raised questions regarding the nature of the inclusion bodies (42). We failed to detect any virus protein sequences in our proteomic analyses. Dietary factors such as calcium and/or vitamin D have also been implicated in disease etiology. The individuals in the Norton Priory collection were likely of a high status based on their distinct and highly decorated stone coffins and prominent burial locations within the priory walls. Stable isotope analysis (C, N) indicate a mainly marine-based diet that was unlikely to be nutrient-deficient, with values similar to King Richard III and the medieval Bishops of Whithorn in Scotland (43, 44). Unknown genetic factors could also have played a role, with recent studies showing variants in genes other than *SQSTM1*, such as zinc finger protein 687 (*ZNF687*), to be associated with severe PDB, with a high number of affected bones; however, in this case, mutations rarely occur in PDB patients without neoplastic degeneration (45). Clearly, other genes interact with *SQSTM1* to modify disease phenotypes, and a more comprehensive understanding of contemporary PDB genetics will direct future studies of ancient forms of the disorder (46).

We propose that, across several centuries of the medieval period, PDB at Norton Priory was very different from the disorder seen in the present day. There are currently uncharacterized environmental factors possibly affecting genetically predisposed individuals to die of a highly unusual variant of the contemporary disease. Identification of these factors could have significant implications for understanding and managing PDB several hundreds of years after the dissolution of the priory.

## Materials and Methods

### Skeletal Remains.

Skeletons are from the Norton Priory Human Skeletal Collection, which is housed at Norton Priory Museum and Gardens in Runcorn, UK. The skeletons are the subject of a new archaeological research framework encouraged by the Trust of Norton Priory Museum and Gardens. The Augustinian Priory was founded in 1115 AD and later relocated to its current location in 1134 AD. Archaeological excavations in the 1970s and 1980s revealed that burials began in the late 12th century and continued until the dissolution of monasteries in 1536 AD. The excavations resulted in the disinterment of 130 articulated skeletons. The deceased are believed to be Augustinian Canons or important benefactors of the priory (47). A recent examination of the collection reports a profile typical of a monastic site, with 85 adult male and 29 adult female burials. The remaining burials are children, inclusive of one fetus found in the abdomen of a female burial. In this study, 18 adult skeletons were investigated in detail (*SI Appendix*, Table S2).

### Contemporary Bone Samples.

Ethical approval to obtain and study human material was granted by the University of East Anglia Faculty of Medicine and Health Sciences Research Ethics Committee (reference 2013/2014–22 HT). Bone samples (*n* = 5) were collected as part of a previous study (17). All individuals provided written informed consent.

### Recombinant Protein Expression in *E. coli.*

GST-tagged human p62 proteins were expressed from plasmid pGEX-4T1 in XL10-Gold *E. coli* (48). Briefly, 10-mL cultures were grown in LB containing 100 µg/mL ampicillin overnight at 37 °C in a shaking incubator. Overexpression cultures (10 mL) were set up by using 200 µL of overnight culture in 10 mL LB containing 100 µg/mL ampicillin. These were grown for 3 h at 37 °C in a shaking incubator and then induced with 400 µM IPTG for a further 3 h.

### Macroscopic Osteological Analysis.

Skeletons (*N* = 18) were examined for distinct visual macroscopic lesions resembling PDB. This included changes in the size, thickness, and vascularity of the bones. A visual inventory was completed for each skeleton. This permitted the documentation of the skeletal distribution of the observed macroscopic changes observed for each individual. One skeleton included in this study (SK29) exhibits marked lesions of an osteosarcoma to both os coxae (20).

### Radiographic Osteological Analysis.

When macroscopic analysis had been completed, skeletons were subjected to radiological review. The radiological features observed in the internal structure of ancient bones with PDB-like changes have been described by several authors and include a lack of uniformity in areas of increased bone density, cortical thickening, and disorganization of the architecture (49). The early lesions of contemporary PDB are predominately lytic and are often limited to the endosteum or central layers of the cortex (50). Radiography permits the detection of earlier phases of PDB that may not be detected by macroscopic analysis alone. Skeletons were subjected to full radiographic review (anteroposteriorly and mediolaterally) using the Faxitron series X-ray system. To document the presentation and distribution of the radiological lesions, a visual inventory was completed for each skeleton.

### AMS Radiocarbon Analysis.

A single tooth was extracted from 15 skeletons. Molar roots were submitted for AMS radiocarbon dating to establish their age and stable isotopic analysis (carbon and nitrogen) for paleodiet reconstruction to Beta Analytic. Molar crowns were submitted to the Natural Environment Research Council Isotope Laboratory (Nottingham, UK) to study their enamel composition to obtain their strontium isotope (^87^Sr/^86^Sr) and oxygen isotope (δ^18^O) values. Teeth were second permanent molars and premolars, as they represent a time frame of 2.5–8.5 y based on the crown formation (51). This analysis was used to estimate the individual’s geographical provenance and potential mobility, depending on the parent rock/soil types where these individuals lived as children (52).

### Protein Extraction from Ancient Bones/Teeth.

Protein was extracted as described previously (53). Sampling preference was for the petrous portion of the temporal bone. In cases in which petrous was not available, teeth were sampled. When teeth were not available, the most intact bone available was sampled (e.g., femur). Samples were cleaned and UV-treated before subsampling was carried out for DNA extraction or protein extraction. Samples were finely ground with a chilled pestle and mortar. Samples were washed for 24 h in 5 volumes (weight to volume) of extraction buffer A (4 M guanidine-HCl, 20 mM NaH_2_PO_4_, 30 mM Na_2_HPO_4_, pH 7.4, plus 1:1,000 mammalian protease inhibitor mixture; Sigma) under constant agitation at 4 °C. Bone was then pelleted by centrifugation at 4 °C, and the supernatant was removed. This was followed by extraction with five volumes of buffer B (buffer A plus 300 mM EDTA) under constant agitation at 4 °C for 24 h. Supernatant was removed as for the previous step. The resultant pellet was washed with five volumes of autoclaved distilled water three times before storage at 20 °C. A total of 20 mg of dried bone matrix extract (BME) was sonicated for 2 × 15 s on ice in 100 µL neutral 50 mM phosphate buffer, 10 mM EDTA, containing 1:1,000 mammalian protease inhibitor mixture. The supernatant was then removed. Solubilized protein was precipitated with TCA (8% wt/vol final concentration). The remaining BME pellet was also retained.

### SDS/PAGE and Western Blotting.

Protein samples were solubilized by using SDS/PAGE loading buffer containing urea (150 mM Tris, 2.5% wt/vol SDS, 3% wt/vol DTT, 10% vol/vol 2-mercaptoethanol, 20% vol/vol glycerol, 8 M urea, 0.1% wt/vol bromophenol blue, pH 6.8). Proteins were separated by gel electrophoresis on 5–20% acrylamide gradient gels with silver staining detection using a GE Healthcare PlusOne silver stain kit or transferred to nitrocellulose for Western blotting. p62-positive control for Western blot was a total protein lysate of the human embryonic kidney cell line HEK293T. A mouse monoclonal p62 antibody (610833; BD Biosciences) and rabbit polyclonal p62 antibody (BML-PW9860; Enzo Life Sciences) were used to probe for p62. HRP-conjugated secondary antibodies were appropriate to primary antibody species (Dako). Secondary antibodies were detected via chemiluminescence using Western Lightning ECL (Perkin-Elmer). Recombinant GST-p62 proteins were similarly detected by SDS/PAGE and Western blotting.

### Recombinant GST-p62 Purification and Western Blotting.

All GST-p62 mutants were produced by site-directed mutagenesis of WT human sequence (1–440) in pGEX-4T-1 using the QuikChange 2 method (Agilent). Bacterial pellets containing expressed proteins were sonicated in 1 mL 10 mM Tris⋅HCl, 150 mM sodium chloride, pH 7.5, 0.1% (vol/vol) Triton X-100 (TBST), followed by centrifugation at 16,000 × *g*, 4 °C for 10 min. The lysates were incubated with 50 µL of preequilibrated glutathione-Sepharose to collect the GST-tagged recombinant proteins. Sepharose pellets were washed three times in TBST, and the proteins were then eluted with SDS/PAGE sample buffer. Samples were resolved on 5–20% acrylamide SDS/PAGE gradient gels and Western-blotted.

### Protein MS.

Complete ancient protein extracts were subjected to LC-MS/MS analysis by electrophoresing the sample ∼0.5 cm in to the resolving gel. After staining (0.1% wt/vol Coomassie blue, 10% vol/vol methanol, 10% vol/vol glacial acetic acid) and destaining (10% wt/vol methanol, 10% vol/vol glacial acetic acid), gel slices containing the protein mixtures were excised. In some cases, gel slices limited to an area including both ancient p62 immunoreactive bands were also analyzed. For shotgun proteomic analysis using the LTQ Orbitrap Velos mass spectrometer (Thermo Fisher Scientific), samples containing protein mixtures were digested with trypsin with subsequent LC-MS/MS analysis using an RSLC nano HPLC system (Dionex) as previously described (54). Raw data files were processed using Proteome Discoverer (v1.4.0.288; Thermo Fisher Scientific) and searched using Mascot (v2.2.04; Matrix Science) against UniprotHuman_2015_02 (67,911 entries) assuming semitrypsin digestion (55). Peptide tolerance was set to 10 ppm, and MS/MS tolerance was set to 0.02 Da. Scaffold Q+S (v4.4.1.1; Proteome Software) was used to validate MS/MS-based peptide and protein identification from Proteome Discoverer (56). Peptide identification was accepted if peptides could be established at >95% probability, with a minimum of two peptides required for protein identification. Targeted LC-MS/MS data of p62-containing gel slices was generated on an Orbitrap Fusion Lumos mass spectrometer following digest of reduced (DTT) and alkylated (iodoacetamide) samples using a standard gel-digestion protocol and elastase to maximize sequence coverage (53, 57). Peptides were separated on a nEASY spray column (Thermo Fisher Scientific) and analyzed on a Dionex Ultimate 3000/Orbitrap Fusion Lumos (Thermo Fisher Scientific) using standard parameters. MS data were analyzed with PEAKS Studio 8.5 (Bioinformatics Solutions). Mass tolerances were set to 10 ppm/0.5 Da (MS1/MS2). No cleavage specificity was defined. Carbamidomethylation was set as fixed. Modification, deamidation, and oxidation were variable. Peptide false discovery rate was adjusted to 1%.

### Sequencing of Ancient DNA.

Bone/tooth samples were cleaned and UV-treated. DNA was extracted by using an optimized protocol developed previously (58). Quantification of each extract and the control ladder was performed on a Bioanalyzer 2100 (Agilent) using a DNA 1000 chip. The efficiency of recovery for each fragment was calculated by dividing the concentration of DNA in the extract by that of the control ladder. DNA was stored at −20 °C. Single-stranded libraries were generated as previously described (59). Libraries were sequenced on a NextSeq 500 (Illumina). Primers used to create the *SQSTM1* amplicon and pigtail primers to allow clearer direct sequencing are detailed in *SI Appendix*, Table S6.

### Sequencing of Ancient Small RNAs.

A bone sample was collected from an osteosarcoma lesion in the pelvis of SK29, with a further sample collected from the femur that was affected by PDB in the same individual. Bone was cleaned and UV-treated before homogenization under liquid nitrogen conditions using the BioPulverizer (BioSpec). Total RNA was extracted by using the miRCURY RNA isolation kit (Exiqon) according to the manufacturer’s instructions. RNA concentration and integrity was measured on a NanoDrop 8000 Spectrophotometer (Thermo Fisher Scientific) and visually assessed by agarose gel electrophoresis with ethidium bromide staining. RNA was stored at −80 °C. Libraries using 5′ and 3′ high definition adapters were generated as previously described (60, 61). Libraries were sequenced on a HiSeq 2500 (Illumina).

### Bioinformatics.

Raw FASTQ files were converted to FASTA format. Reads containing unassigned nucleotides were excluded. The 3′ adapter was trimmed by using perfect sequence similarity to the first 8 nt of the 3′ HiSeq 2500 adapter (TGGAATTC). The high definition signatures (four assigned degenerate nucleotides at the ligating ends) of the reads were also trimmed. SRNAs were mapped full length with no mismatches to the human genome (v38) and corresponding annotations using PatMaN (62). The latest set of human miRNAs were downloaded from miRBase (v21) (63). Normalization and differential expression analysis was performed by using DESeq2 (v1.2.10) (64). Independent filtering was used to remove low-expressing transcripts in normalized counts. miRNAs were considered differentially expressed if they had a *P* value <0.05, <5% false discovery rate according to the Benjamini–Hochberg procedure, and greater than twofold change in expression.

### Digital PCR.

Total RNA was quantified by density measurement after separation by agarose gel electrophoresis with ethidium bromide staining. Equal amounts of RNA were reverse-transcribed using the TaqMan advanced miRNA cDNA synthesis kit (Thermo Fisher Scientific). Differential expression of miR-16 was validated in triplicate by using TaqMan miRNA advanced assays (Thermo Fisher Scientific). Digital PCR was performed on the QuantStudio 3D Digital PCR System using the GeneAmp PCR System 9700 (Thermo Fisher Scientific). After PCR, the chips were imaged on the QuantStudio 3D Instrument, which assesses the raw data and calculates the concentration of the cDNA sequence targeted by FAM- and VIC-labeled probes by Poisson distribution (65). For more in-depth analysis, the QuantStudio 3D AnalysisSuite was used to report the data as copies per microliter. The probe sequence used was miR-16–5p (5′-UAGCAGCACGUAAAUAUUGGCG-3′).

### Data Availability.

Proteomic data are available on ProteomeXchange under accession number PXD011743 (66). Sequencing data are available on the Gene Expression Omnibus database under accession numbers GSE87021 and GSE118540 (67).

## Supplementary Material

Supplementary File
