# Supplementary material for: Molecular insights into an ancient form of Paget’s disease of bone
Source: Proc Natl Acad Sci U S A. 2019 Apr 29;116(21):10463–72. doi: 10.1073/pnas.1820556116 (PMC6535003; doi:10.1073/pnas.1820556116)
Supplement: Supplementary File [file pnas.1820556116.sapp.pdf]

PNAS
www.pnas.org

Supplementary Information for

Molecular insights into an ancient form of Paget's disease of bone

Barry Shaw, Carla L Burrell, Darrell Green, Ana Navarro-Martinez, Daniel Scott, Anna Daroszewska, Rob van 't Hof, Lynn Smith, Frank Hargrave, Sharad Mistry, Andrew Bottrill, Benedikt Kessler, Roman Fisher, Archana Singh, Tamas Dalmay, William D Fraser, Kirstin Henneberger, Turi King, Silvia Gonzalez, Robert Layfield

Robert Layfield
Email: robert.layfield@nottingham.ac.uk

This PDF file includes:

Figs. S1 to S2
Tables S1 to S6
References for SI reference citations

www.pnas.org/cgi/doi/10.1073/pnas.1820556116

a

1 MASLTVKAYL LGKEDAAREI RR**FSFCCSPE** **PEAEAEAAAG** **PGPCER**LLSR 50
51 VAALFPALRP GGFQAHYRDE DGDLVAFSSD EELTMAMSYV KDDIFRIYIK 100
101 EKKECR**RDHR** **PPCAQEAPR**N MVHPNVICDG CNGPVVGTRY KCSVCPDYDL 150
151 CSVCEGKGLH RGHTKLAFPS PFGHLSEGFS HSRWLRKVKH GHFGWPGWEM 200
201 GPPGNWSPRP PR**AGEARPGP** **TAESASGPSE** **DPSVNFLK**NV GESVAAALSP 250
251 LGIEVDIDVE HGGKRSR**LTP** **VSPESSSTEE** **K**SSSQPSSCC SD**PSKPGGNV** 300
301 **EGATQSLAEQ** **MR**KIALESEG RPEEQMESDN CSGGDDDWTH LSSKEVDPST 350
351 GELQSLQMPE SEGPSSLD**PS** **QEGPTGLK**EA ALYPHLPPEA DPRLIESLSQ 400
401 MLSMGFSDEG GWLTRLLQTK NYDIGAALDT IQYSKHPPPL 440

b

1 MASLTVKA**YL** **LGKEDAAREI** RRFSFCCSPE PEAEAEAAAG PGPCERLLSR 50
51 V**AALFPALRP** **GGFQAHYRDE** **DGDLVAFSSD** **EELTMAMSYV** **KDDIFRI**YIK 100
101 EKKECRRDHR PPCAQEAPRN MVHPNVICDG CNGPVVGT**RY** **KCSVCPDYDL** 150
151 **CSV**CEGKGLH RGHTKLAFPS PFGHLSEGFS HSRWLRKVKH GHFGWPGWEM 200
201 GPPGNWSPRP PR**AGEARPGP** **TAESASGPSE** **DPSVNFLKNV** **GESVAAA**LSP 250
251 LGIEV**DIDVE** **HGGKRSRLTP** **VSPESSSTEE** **KSSSQPS**SCC SDPSKPGGNV 300
301 EGATQSLAEQ MRKI**ALESEG** **RPEEQMESDN** CSGGDDDWTH LS**SKEVDPST** 350
351 **GELQSLQMPE** **SEGPSSLDPS** **QEGPTGLKEA** **ALYPHLPPEA** **DPRLI**ESLSQ 400
401 MLSMGFSDEG GWLTRLLQTK NYDIGAALDT IQYSKHPPPL 440

Fig. S1. Mass spectrometry data indicating wild type ancient human p62 protein sequence detected (shaded) from SK101 petrous (insoluble pellet) fraction. a Tryptic digestion and analysis using the LTQ Orbitrap Velos. b Elastase digestion and analysis using the Orbitrap Fusion Lumos.

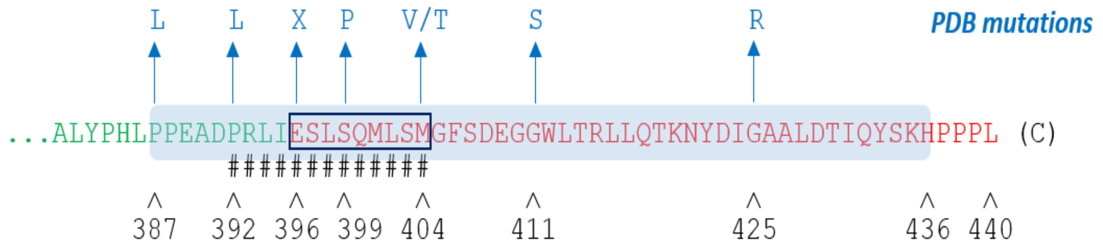

Fig. S2. Schematic representation indicating ancient p62 protein sequence detected (green) or not observed (red) by mass spectrometry. Boundaries of the UBA domain (grey shading, residues 387-436) epitope recognised by the rabbit anti-p62 antibodies (boxed),

Table S1. Skeletal elements with PDB like pathological changes in six skeletons from the Norton Priory collection.

Skeleton (Grave number)	Age-at-death estimation	Skeletal elements affected	
		#Boyleston and Ogden 2005[1]	$Re-analysis
SK22(Gr21)	50-59 years	Cranium, Clavicle (B), Scapula (B), Spine, Humerus (R), Ribs (B), Pelvis (R, S), Femur (B)	Cranium, Clavicle (B), Scapula (B), Spine, Humerus (B), Ulna (B), Radius (B), Metacarpals and phalanges (B), Ribs (B), Pelvis (B, S), Femur (B), Tibia (B), Fibula (B), Tarsals (B)
SK29(Gr28)	45-49 years	Ribs (B), Pelvis (L)	Clavicle (B), Humerus (B), Ulna (L), Radius (L), Metacarpals (B), Ribs (B), Pelvis (B), Femur (B), Tibia (B), Fibula (R)
SK35(Gr34)	50-59 years	Pelvis (L)	Scapula (R), Humerus (R), Pelvis (L), Femur (R)
SK52(Gr50)	50-59 years	Clavicle (L), Scapula (L), Spine, Pelvis (L, S), Femur (R)	Cranium, Clavicle (B), Scapula (B), Spine, Humerus (B), Radius (B), Metacarpals and phalanges (B), Ribs (B), Pelvis (B, S), Femur (B), Tibia (B), Fibula (L), Tarsals (B)
SK55(Gr53)	40-44 years	Clavicle (L), Scapula (L), Spine, Pelvis (L, S), Femur (L), Tibia (L)	Clavicle (L), Scapula (L), Spine, Humerus (L), Ulna (B), Radius (B), Hand phalanges (L), Ribs (B), Pelvis (B, S), Femur (B), Tibia (B), Tarsals (B)
SK101(Gr116)	45-49 years	Cranium, Spine, Humerus (B), Pelvis (B, S), Femur (B), Tibia (B)	Cranium, Clavicle (B), Scapula (B), Spine, Humerus (B), Ulna (B), Radius (B), Metacarpal and phalanges (B), Ribs (B), Pelvis (B, S), Femur (B), Tibia (B), Fibula (B), Metatarsals (B)

#lesions identified by macroscopic analyses
$lesions identified by a combination of radiographic and macroscopic analyses
L=Left, R=Right, B=Both L and R elements, and S=Sacrum

Table S2. AMS radiocarbon analysis of teeth samples taken from 18 skeletons with a PDB like disorder at Norton Priory.

Skeleton number	Sex Assessment	Age-at-death Assessment	Lab Number	AMS Radiocarbon Date 2 Sigma Calibration
SK21(Gr20)	Male	35-39 years	BETA-471835	AD1270-1316 & 1354-1390
SK22(Gr21)	Male	50-59 years	BETA-425284	AD 1050-1080 & AD 1150-1250
SK27(Gr26)	Male	35-39 years	BETA-471836	AD 1396-1445 & AD1328-1341
SK28(Gr27)	Male	50-59 years	BETA-471837	AD1295-1404
SK29(Gr28)	Male	45-49 years	-	Unsuccessful
SK32(Gr31)	Male	45+ years	BETA-471838	AD 1024-1155
SK35(Gr34)	Male	50-59 years	BETA-425286	AD 1155-1260
SK37(Gr35)	Male	35-39 years	BETA-471839	AD1392-1443 & AD 1324-1345
SK42(Gr40)	Male	40-44 years	BETA-471840	AD 1024-1155
SK47(Gr45)	Male	45-49 years	BETA-471841	AD 1150-1256 & AD 1049-1084
SK52(Gr50)	Male	50-59 years	-	No dentition present
SK55(Gr53)	Male	40-44 years	-	Unsuccessful
SK62(Gr64)	Female	40-44 years	BETA-471842	AD 1020-1155
SK78(Gr85)	Female	45-49 years	BETA-471843	AD 1154-1265 & AD 1059-106
SK87(Gr99)	Female	45-49 years	BETA-471844	AD 1292-1400
SK89(Gr101)	Male	45-49 years	BETA-471845	AD1150-1256 & AD 1049-1084
SK94(Gr108)	Male	35-39 years	BETA-471846	AD 1414-1479
SK101(Gr116)	Male	45-49 years	BETA-425288	AD 1280-1320 & AD 1350-1390

Table S3. Stable isotopic analysis of teeth samples taken from 18 skeletons with a PDB like disorder at Norton Priory.

Skeleton Number	C	N	Sr	O
	$\delta^{13}C$	$\delta\ ^{15}N$	$^{87}Sr/^{86}Sr$	$\delta\ ^{18}0\ _{VSMOW}$
SK21(Gr20)	-19.1‰	13.4‰	0.71176	18.4
SK22(Gr21)	-19.3‰	13.3‰	0.71123	18.0
SK27(Gr26)	-18.8‰	11.7‰	0.71106	18.8
SK28(Gr27)	-18.2‰	15.1‰	0.71037	18.0
SK29(Gr28)	-	-	0.71035	18.6
SK32(Gr31)	-19.0‰	12.9‰	0.71087	18.3
SK35(Gr34)	-19.0‰	13.0‰	0.71065	18.6
SK37(Gr35)	-19.1‰	14.1‰	0.71044	18.3
SK42(Gr40)	-19.2‰	13.2‰	0.71051	18.1
SK47(Gr45)	-18.6‰	13.1‰	0.71051	18.1
SK52(Gr50)	-	-	-	-
SK55(Gr53)	-	-	0.71026	18.5
SK62(Gr64)	-18.8‰	13.5‰	0.71004	19.2
SK78(Gr85)	-18.9‰	14.3‰	0.71070	19.0
SK87(Gr99)	-19.1‰	13.6‰	0.71001	18.2
SK89(Gr101)	-19.9‰	12.4‰	0.70975	19.4
SK94(Gr108)	-19.7‰	11.6‰	0.71286	18.7
SK101(Gr116)	-19.2‰	13.2‰	0.71038	18.4

The mean ratio of δ13C is -19.1‰ and for δ15N is 13.2‰, these values are elevated above what would be expected from a purely terrestrial diet, identifying a contribution from marine (freshwater fish). The $^{87}Sr/^{86}Sr$ ratios cover a broad range from 0.70975 to 0.71286, when they are compared against a Sr isotopic map the ratios indicate association with rocks/soils found in the North West and the Western part of the UK. The values for $\delta^{18}O$ range from 18.0 to 19.4. Due to variation in rainfall levels, the mean $\delta^{18}O$ value for the Western UK is 18.2% +/- 3 and for the Eastern UK the mean value is 17.2% +/- 1.3. Results are consistent with a North Western UK origin.

Table S4. Skeletal distribution of the PDB like disorder in the 18 skeletons from Norton Priory (left and right sides combined).

**Area affected**	**Percentage at Norton Priory (n=18)**	
	**(Macroscopic results)**	**(Radiographic results)**
Skull	77.7%	94.4%
Clavicle	44.4%	72.2%
Scapula	27.7%	77.7%
Humerus	50.0%	94.4%
Ulna	55.5%	94.4%
Radius	27.7%	94.4%
Hand	16.7%	38.9%
Cervical spine	22.2%	33.3%
Thoracic spine	22.2%	33.3%
Lumbar spine	27.7%	33.3%
Pelvis	44.4%	77.7%
Sacrum	27.7%	44.4%
Femur	44.4%	77.7%
Tibia	50.0%	50.0%
Fibula	61.1%	88.9%
Feet	27.7%	44.4%

Table S5. Human proteins and their accession numbers identified from the petrous bone of SK101 by mass spectrometry.

**Protein Name**	**Protein Accession Number**	**[#]Coverage %**	**[$]Unique peptides**
Collagen alpha-2(I) chain OS=Homo sapiens GN=COL1A2 PE=1 SV=1	A0A087WTA8, P08123	17	39
Collagen alpha-1(I) chain OS=Homo sapiens GN=COL1A1 PE=1 SV=5	P02452	10	17
Keratin, type I cytoskeletal 10 OS=Homo sapiens GN=KRT10 PE=1 SV=6	P13645	41	17
Keratin, type II cytoskeletal 2 epidermal OS=Homo sapiens GN=KRT2 PE=1 SV=2	P35908	40	15
Keratin, type II cytoskeletal 1 OS=Homo sapiens GN=KRT1 PE=1 SV=6	P04264	31	14
Keratin, type I cytoskeletal 9 OS=Homo sapiens GN=KRT9 PE=1 SV=3	P35527	25	11
Vitronectin OS=Homo sapiens GN=VTN PE=1 SV=1	P04004	20	10
Alpha-2-HS-glycoprotein OS=Homo sapiens GN=AHSG PE=1 SV=1	C9JV77, P02765	20	9
Thrombospondin-1 OS=Homo sapiens GN=THBS1 PE=1 SV=2	P07996	3	4
Prothrombin OS=Homo sapiens GN=F2 PE=1 SV=2	P00734	8	4
C-type lectin domain family 11 member A OS=Homo sapiens GN=CLEC11A PE=1 SV=1	A0A0A6YY95, Q9Y240	11	4
**Sequestosome-1 OS=Homo sapiens GN=SQSTM1 PE=1 SV=1**	**Q13501**	**8**	**3**
Collagen alpha-1(II) chain OS=Homo sapiens GN=COL2A1 PE=1 SV=3	P02458	2	3
Collagen alpha-2(XI) chain OS=Homo sapiens GN=COL11A2 PE=1 SV=1	A0A0C4DFS1, A0A0G2JL35, P13942	2	3
Serum albumin OS=Homo sapiens GN=ALB PE=1 SV=2	P02768	3	2
Keratin, type II cytoskeletal 6A OS=Homo sapiens GN=KRT6A PE=1 SV=3	P02538	12	1
Keratin, type I cytoskeletal 14 OS=Homo sapiens GN=KRT14 PE=1 SV=4	P02533	11	1
Keratin, type I cytoskeletal 28 OS=Homo sapiens GN=KRT28 PE=1 SV=2	Q7Z3Y7	8	1
Keratin, type II cytoskeletal 5 OS=Homo sapiens GN=KRT5 PE=1 SV=3	P13647	10	1

[#]primary sequence coverage for the particular protein
[$]exclusive unique peptide counts

Table S6. Primer sequences used to create the ancient *SQSTM1* amplicon (all 5’-3’).

SQSTM1_F	GGCTTCCTTACTGTTTCGGC
SQSTM1_R	CCATGGACAGCATCTGGGAG
SQSTM1Tail_F	AACTGACTAAACTAGGTGCCACGTCGTGAAAGTCTGACAAGGCTTCCTTACTGTTTCGGC
SQSTM1Tail_R	AACTGACTAAACTAGGTGCCACGTCGTGAAAGTCTGACAACCATGGACAGCATCTGGGAG

References

1. Boylston, A. & Ogden, A. ‘A study of Paget’s disease at Norton Priory, Cheshire, a Medieval religious house’ [in] *Proceedings of the Fifth Annual Conference of the British Association for Biological Anthropology and Osteoarchaeology*. Vol. British Archaeological reports (eds. Zakrzewski, S.R., Clegg, M., British Association for Biological, A. & Osteoarchaeology, C.) 69-76 (Archaeopress, Oxford, 2005).
